# Meiotic Chromosome Analysis of the Giant Water Bug, *Lethocerus indicus*

**DOI:** 10.1673/031.013.3901

**Published:** 2013-05-13

**Authors:** Wijit Wisoram, Pradit Saengthong, Lertluk Ngernsiri

**Affiliations:** Department of Genetics, Faculty of Science, Kasetsart University, Bangkok, Thailand.

**Keywords:** chromosome univalent, holocentric chromosome

## Abstract

The giant water bug, *Lethocerus indicus* (Lepeletier and Serville) (Heteroptera: Belostomatidae), a native species of Southeast Asia, is one of the largest insects belonging to suborder Heteroptera. In this study, the meiotic chromosome of *L. indicus* was studied in insect samples collected from Thailand, Myanmar, Loas, and Cambodia. Testicular cells stained with lacto-acetic orcein, Giemsa, DAPI, and silver nitrate were analyzed. The results revealed that the chromosome complement of *L. indicus* was 2n = 22A + neo-XY + 2m, which differed from that of previous reports. Each individual male contained testicular cells with three univalent patterns. The frequency of cells containing neo-XY chromosome univalent (∼5%) was a bit higher than that of cells with autosomal univalents (∼3%). Some cells (∼0.5%) had both sex chromosome univalents and a pair of autosomal univalents. None of the m-chromosome univalents were observed during prophase I. In addition, this report presents clear evidence about the existence of m-chromosomes in Belostomatidae.

## Introduction

Giant water bugs are the largest insects distributed worldwide. Their body length can be up to 12 cm. The insects belong to the family Belostomatidae, which has three subfamilies, Belostomatinae, Horvathininia, and Lethocerinae (Lauck and Menke 1961; Perez-Goodwyn 2006). The last subfamily consists of three genera, *Lethocerus* Mayr, *Kirkaldyia* Montandon, and *Benacus* Stal. The giant water bug belongs to the genus *Lethocerus*. Until now, 22 species of giant water bug have been identified. Of these 22 species, 16 species are found in the American continent and six species are distributed throughout the rest of the world (Perez-Goodwyn 2006). *Lethocerus indicus* is the native giant water bug of Southeast Asia. In Thailand, people have used the male bug as an aromatic ingredient in some native curry pastes. The fragrance is a sex pheromone produced by male bugs to attract the females (Butenandt and Tarn 1957; Devakul and Maarse 1964). Because of an increasing demand of its pheromone, unsuccessful attempts to rear it, and changes in its environment, the number of *L. indicus* in nature in Thailand has gradually decreased. As a result, they have been imported from neighboring countries such as Cambodia and Loas.

The cytogenetics of Heteropteran insects are interesting primarily because they possess holocentric chromosomes. The chromosomes do not have a localized centromere, but the centromere is distributed along the length of the chromosome (Ueshima 1979). Due to this characteristic, if a chromosome is broken, the fragments are not lost and still move to a pole at anaphase (Hughes-Schrader and Schrader 1961; LaChance et al. 1970). Moreover, the meiotic behavior of autosomes and sex chromosomes are different. As a rule, autosomes form bivalents with one chiasma per bivalent and divide pre-reductionally, while sex chromosomes are achiasmatic and form univalents at the first meiosis. The sex Chromosoms divide equationally at anaphase I and segregate reductionally at anaphase II (Ueshima 1979; Rebagliati et al. 2005). Moreover, some families also possess a pair of m-chromosomes, which are also achiasmatic. The mchromosomes are unpaired and present as univalent chromosomes during early meiosis, but at metaphase I they form a pseudobivalent and divide reductionally at meiosis I and segregate equationally at meiosis II. Four sex chromosome systems have been described in 1600 species of Heteroptera. The XX/XY system is the most commonly found (71.4%), the XO/XX and multiple system (X_n_Y/X_n_X_n_, X_n_O/X_n_X_n_, XY_n_/XX) are found in 14.7% and 13.5% of species respectively, and the rare system (0.5%) is the neo-sex chromosome system that has been reported in seven species, including *Lethrocurus indicus* (Grozeva and Nokkala 1996; Bressa et al. 1999; Nokkala and Nokkala 1999; Jacobs 2004; Papeschi and Bressa 2006)

The cytogenetics of Belostomatidae have been revealed by the studies on seventeen *Belostoma* species, three *Diplonychus* species, and seven *Lethocerus* species (Papeschi and Bressa 2006). In these *Lethocerus* species, their chromosome numbers vary. Three species, *L. annulipes, L. griseus*, and *L. melloleitaoi* De Carlo, contain the same chromosome complement, 2n = 26A + XY, while the chromosome complement of *L. indicus* is 2n = 24A + neoX-neoY. It has been suggested that the neoX and neoY chromosomes are established by the translocation of X and Y chromosomes to one pair of autosomes (Nokkala and Nokkala 1999). The chromosome number is quite reduced in *L. americanus* Leidy (2n = 6A + XY) and *Lethocerus* sp. (2n = 2A + neoX-neoY), while it is increased in *L. uhleri* (Montandon) (2n = ca. 30). However, the chromosomal behavior during spermatogenesis of the *Lethocerus* species has not been described, except the chromosome formula, because the cytogenetics of most species were studied during 1927 and 1959, and the original papers are difficult to access (Papeschi and Bidau 1985; Papeschi and Bressa 2006).

In the present study, the chromosome complement and the behavior of meiotic chromosomes of *L. indicus* from Thailand, Loas, Myanmar, and Cambodia were studied using lacto-acetic orcein squash technique, Giemsa, DAPI, and silver staining. The results showed that the chromosome complement of *L. indicus* revealed in this study differed from that previously reported, and also presented clear evidence of the existence of mchromosomes in the Belostomatidae family. The presence of chromosome univalents and the behavior of chromosomes during spermatogenesis were also discussed.

## Materials and Methods

### Insects

Living adults of *L. indicus* were collected from natural populations in three provinces of Thailand, namely Chiang Mai, Buri Ram, and Sa kaeo. For samples from Myanmar, Laos, and Cambodia, the living insects were bought from importing merchants at markets near the borders of Thailand.

### Slide preparations

The testes of *L. indicus* were dissected and fixed in ethanol:acetic acid (3:1) overnight at 4° C. Then, they were stored in 70% alcohol at 4° C until used. For standard staining, the fixed testes stained with lacto-acetic orcein were squashed on slides. This technique was used to examine chromosome types in all insect populations.

For Giemsa, DAPI, and silver nitrate stainings, the fixed testicular cells were dissociated in a few drops of 60% acetic acid for 5 min, spread on slides, placed on a slide warmer at 50° C, and kept at room temperature for further staining. The slides were stained with 4% Giemsa (Merck, www.merck.com) in phosphate buffer, pH 6.8, for 30 min, rinsed thoroughly with distilled water, and then airdried. Silver nitrate staining was performed according to the protocol of Howell and Black (1980). Briefly, two solutions were prepared, one was colloidal developer solution (2 g powder gelatin, 1 mL formic acid in 100 mL distilled water) and the other was silver nitrate solution (4 g AgN03 dissolved in 8 mL distilled water). For staining, 200 µl of colloidal developer solution was mixed with 400 µl of silver nitrate solution and then dropped on the slides, covered with coverslides, and placed on a slide warmer at 70° C for 2 min. Then, distilled water was used to remove the coverslides and excess staining mixture. The stained slides were air-dried and examined. All slides stained with Giemsa and silver nitrate were observed under a B×41 Olympus microscope (www.olympus-global.com) using an immersion oil objective (100×) and all photographs were taken with an Olympus DP72 digital camera. For DAPI staining, DAPI (4-6-diamidino-2-phenylindole; Sigma-Aldrich, www.sigmaaldrich.com) was dissolved in distilled water to get 1 µg/ml. The DAPI solution was dropped on the slides and covered with coverslides. The slides were then observed immediately under a Zeiss fluorescence microscope (www.zeis.com) with a 100× objective, and the images were captured by the Isis FISH imaging system version 5.2 (www.isisimaging.com).

**Figure 1.**
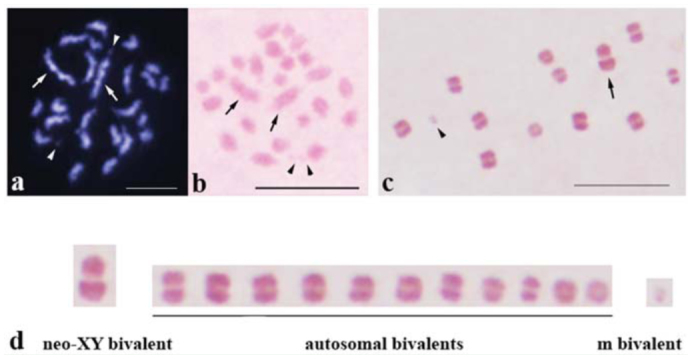
Male meiosis in *Lethocerus indicus* (2n = 22 A + neo-XY + 2m). (a) Mitotic prometaphase of spermatogonia stained with DAPI and (b) stained with lacto-acetic orcein. Arrows point to sex chromosomes and arrowheads point to m-chromosomes. (c) Metaphase 1 chromosomes stained with lacto-acetic orcein and (d) karyotype with 11 autosomal bivalents, a neo-XY bivalent, and an m-chromosome bivalent. Bar = 10 µm. High quality figures are available online.

## Results

### Chromosome complement

In this study, the behavior of chromosomes during spermatogenesis in *L. indicus* was observed. The mitotic metaphase of *L. indicus* spermatogonia was comprised of 26 chromosomes. The two large chromosomes and two micro (m) chromosomes were obvious. It was difficult determine whether the remaining chromosomes were of middle or small size. Therefore, it was also quite difficult to perform a karyotype from mitotic chromosomes (Figure 1a, b). The chromosome complement of *L. indicus* inferred from those in meiotic testicular cells at diakinesis and metaphase I stages was 2n = 22 + neoX-Y + 2m (Figure 1c). The meiotic karyotype was comprised of 13 bivalents: one large, nine medium, two small, and one micro (Figure 1d).

### Meiotic chromosome behavior

**Figure 2.**
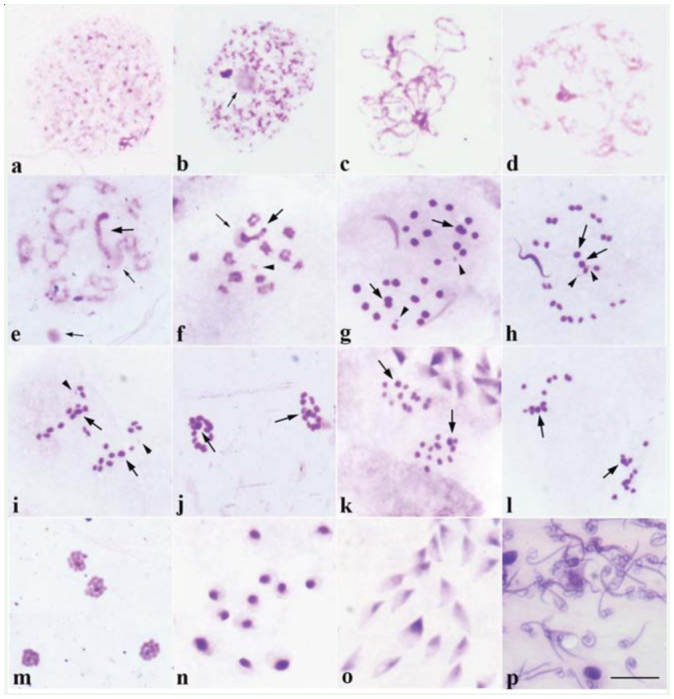
Meiotic chromosome behavior during *Lethocerus indicus* spermatogenesis stained with Giemsa. (a) polyploid nutritive cells with many heteropicnotic chromatin bodies; (b) leptotene-zygotene; (c) pachytene; (d) diffuse stage; (e) diplotene; (f) diakinesis; (g) metaphase I; (h, i) anaphase I; (j) telophase I;. (k) metaphase II; (I) anaphase II; (m) telophase II; (n) round spermatids; (o) elongating spermatids; (p) head of spermatozoas. Big arrows indicate sex chromosomes, small arrows point to nucleoli and arrowheads indicate m-chromosomes. Bar = 10 µ. High quality figures are available online.

**Figure 3.**
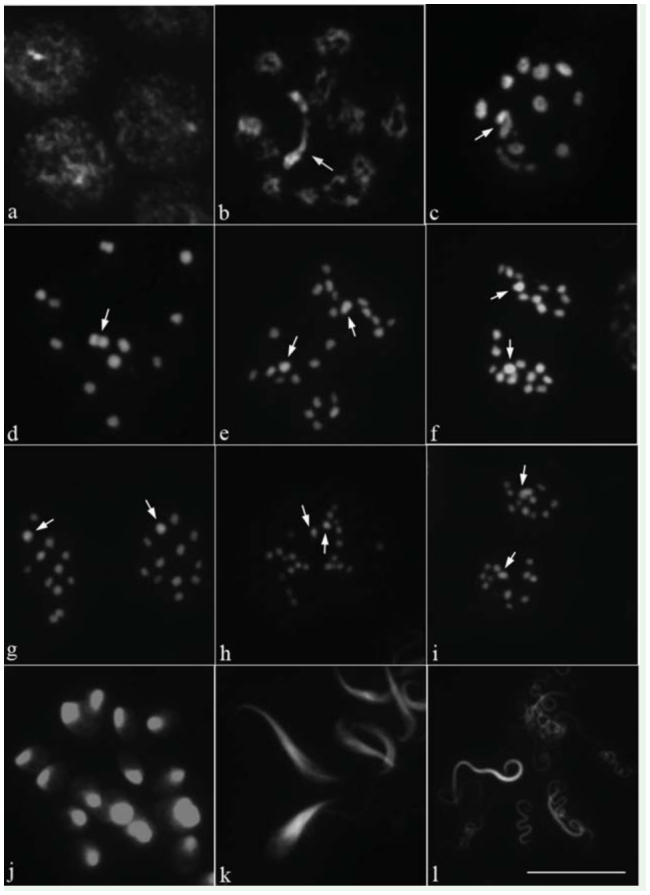
Meiotic chromosome behavior during *Lethocerus indicus* spermatogenesis stained with DAPI. (a) leptotene; (b) diplotene; (c) diakinesis; (d) metaphase I; (e) anaphase I; (f) telophase I; (g) metaphase II; (h) anaphase II; (i) telophase II; (j) early spermatids; (k) elongating spermatids; (I) head of spermatozoas. Arrows indicate sex chromosomes. Bar = 10 µm. High quality figures are available online.

The behavior of meiotic chromosomes was described in a typical cell. The polyploidy nuclei of nutritious cells at the seminiferous tubules walls had several heteropycnotic bodies with similar size contributing evenly (Figure 2a). In leptotene, the sex chromosomes appeared in one or two positively heteropycnotic bodies associated with the chromatin threads, while the nucleolus was less positively stained (Figure 2b). The chromosome threads were clearly seen in pachytene (Figure 2c). In diffuse stage, the heteropycnotic bodies were still obviously seen, while autosomes were partially decondensed, and the less heteropycnotic positive nucleolus was round in shape (Figure 2d, 3a). At diplotene, 11 autosomal bivalents formed, and most bivalents or sometimes all bivalents appeared as rings. The ring bivalent indicated that a bivalent had two terminal chiasmata, while the neo-XY chromosomes formed a bivalent with terminal association and were always associated with the nucleolus (Figure 2e, f, 3b). At diakinesis, the neo-XY chromosome could be seen conspicuously, with both ends staining darker than the remaining part, and the m-chromosomes could also be seen (Figure 2f, 3c). In some cells, two round nucleoli were observed. One was always associated with the neo-XY chromosome, and the other one could be located either close or far from the chromatin (Figure 2e). At late diakinesis, most bivalents appeared in short rod shapes, because one chiasma of the ring bivalent was released so that they became rod bivalents with terminal association, but some autosomal bivalents were still present in rings (Figure 1c). At metaphase I, nucleoli disappeared. The arrangement of bivalents did not form a particular pattern. However, in most cells, the neo-XY bivalent and one or two autosomal bivalents were surrounded by other autosomal bivalents (Figure 2g, 3d). The neo-XY bivalent was the biggest and was heteromorphic, comprising two chromosomes, one of which was a bit bigger than the other, associated together. The bigger one was probably the neo-X and the smaller might have been the neo-Y (Figure 1d, 2g, 3d). The autosomal bivalents were slightly different in size. From diplotene to telophase II, the mchromosomes were always obviously seen as the smallest bivalent (Figure 2f–h). All bivalents showed axial orientation in the spindle. At anaphase I, all bivalents divided reductionally (Figure 2h, i, 3e, f). Therefore, at telophase I, two daughter nuclei had a different sex chromosomes because one received the neo-X and the other obtained the neo-Y. However, it was impossible to identify which one received neo-X or neo-Y, since the size of both sex chromosomes was only slightly different (Figure 2i, 3e). Then, the chromosomes congregated together, and the sex chromosome was obviously seen, with darker staining and the biggest size in the center (Figure 2j, 3f). In most meiosis II cells, metaphase II chromosomes had a radial configuration in which autosomes formed a ring with the sex chromosome as a part of the ring (Figure 2k, l, 3g). At anaphase II, all chromosomes divided equationally, and all chromosomes congregated together with the sex chromosome, neoX or neo-Y, obviously seen in the center at telophase II (Figure 2m, 3h). The spermiogenesis process began with a round spermatid containing a round, highly-stained, heteropycnotic body at the periphery or center (Figure 2n, 3j). Subsequently, the shape of spermatids elongated, and they could be seen as isosceles triangle-like with the sharpest corner being heteropycnotic (Figure 2n, 3h). When the spermatid differentiated further, its shape became longer and the head of the spermatozoa was strongly stained (Figure 2p, 3l).

### Univalent chromosomes

**Figure 4.**
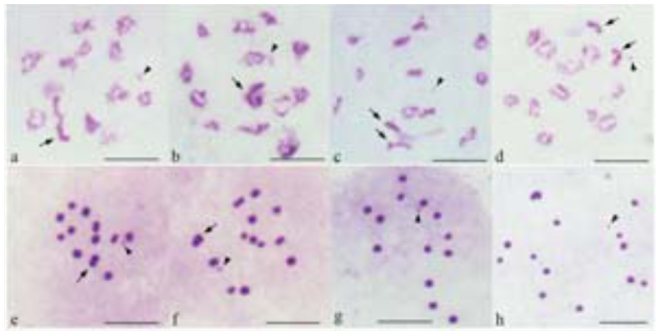
Four testicular cell types found in each testis of *Lethocerus indicus*. (a, e) A typical cell type, type A cell have no univalent; (b, f) Type B cells with two autosomal univalents; (c, g) Type C cells with two sex chromosome univalents; (d, h) Type D cells with four univalent, two autosomal and sex chromosome univalents. Arrows indicate neo-XY chromsomes and arrowhead point to m-chromosomes. Bar = 10µm. High quality figures are available online.

In the testis of an individual male, four cell types, designated A, B, C and D, with different patterns of chromosome univalents were observed. Type A cells with no univalent were the typical chromosome pattern of testicular cells of *L. indicus* in this study. Type A cells were present in majority (∼92%), comprised of 13 bivalents, including the neo-XY chromosome bivalent, which was the largest bivalent, 11 autosomal bivalents, which were similar in size, and an m-chromosome bivalent (Figure 4a, e). The other three cell types were characterized by the presence of chromosome univalents. Type B cells contained 12 bivalents (10 autosomal bivalents, one sex chromosome bivalent, and an m-chromosome bivalent) and two autosomal univalents (Figure 4b,f). Type C cells also possessed 12 bivalents (11 autosomal bivalents and an mchromosome bivalent) and two sex chromosome univalents, neo-X and neo-Y (Figure 4c, g). The frequency of type C cells (∼5%) was a bit higher than that of type B cells (∼3%). Some cells (∼0.5%) were in type D, with 11 bivalents and four chromosomal univalents as the result of both the sex chromosomes and a pair of autosomes forming univalents (Figure 4d, h). Because autosomes were similar in size, it was impossible to identify with certainty which autosomal pair formed univalents, and whether autosomal univalents were present in cells . The frequency of each cell type in each population is presented in Table 1.

### DAPI staining

The testicular cells of *L. indicus* were stained with DAPI with the expectation that some fluorescent banding patterns would be observed; there was this expectation because DAPI stains AT-rich DNA in chromosomes. In mitotic chromosome (Figure 1a), all chromosomes contained DAPI-bright bodies. Conspicuously, the two longest chromosomes, neo-X and neo-Y, had three DAPI-bright bodies, two big bodies, and a small one. The patterns of the DAPI-bright bodies in the two sex chromosomes were different. One sex chromosome contained a big bright, body at the end of chromosome, followed by another big one and then a small one near the middle of the chromosome. In the other sex chromosome, two big, bright bodies were located close to the middle, and the small one was next to one of the big bodies (Figure 1a). All autosomes contained one or two DAPI-bright bodies, while m-chromosomes showed relatively less brightly. In meiotic cells, one or two DAPI-bright bodies were found in a cell at the early stage of meiosis, leptotene (Figure 3a). At diplotene, a neo-XY chromosome was clearly distinguished from all autosomal bivalents and showed obviously DAPI-bright bodies at both chromosome ends. One end was bigger and brighter than the other, which displayed two DAPI-bright bodies (Figure 3b). From diakinesis onwards, all chromosomes showed almost the same level of DAPI-brightness except sex chromosomes, which displayed a bit brighter (Figure 3c–i). At the early spermiogenesis, each round spermatid contained a DAPI-bright body at one peripheral side (Figure 3j). Later, spermatids were elongated, and a DAPI-positive signal had still been at their sharp corners (Figure 3k). In mature spermatozoa, the positive DAPI signals were present only in their heads (Figure 3l).

**Figure 5.**
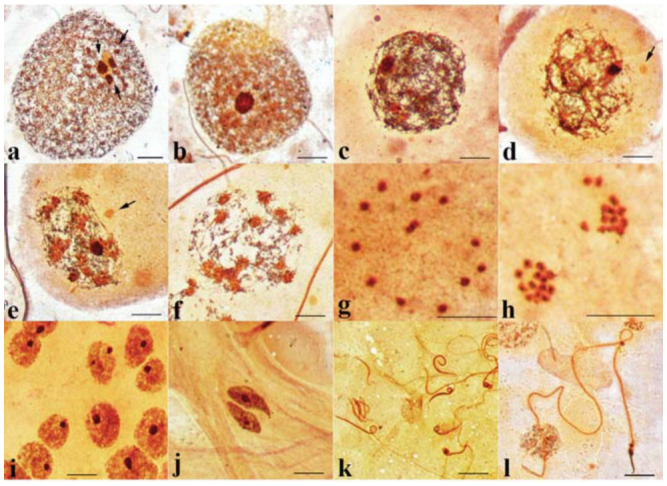
Silver nitrate staining the testicular cells of *Lethocerus indicus*. (a) polyploid nutritive cell showing two impregnated regions (arrows), (b) early leptotene with one nucleolar body; (c) zygotene; (d) pachytene; (d, e) arrows point to the nucleolus located out off chromatin region; (e, f) diplotene; (g) metaphase I; (h) telophase I; (i) round spermatids with nucleolar bodies at the periphery; (j) elongating spermatids with nucleolar bodies at the center of the heads; (k–l) mature spermatozoa showing the heads strongly impregnated with silver nitrate. Bar = 10 µm. High quality figures are available online.

### Silver staining

Silver nitrate has been used to reveal nucleolus and nucleolar organizing regions on chromosomes of many insect species, including Heteropteran insects. In this study, the nutritive polyploidy cells of testes stained with silver nitrate showed one or two impregnated regions of nucleolar material (Figure 5a). Spermatogonial cells at early prophase I showed one or two nucleoli (Figure 5b, c). Most testicular cells at leptotene-pachytene contained two nucleolar bodies, one with strong staining associated with the chromatin, and another one with less staining located far from the chromatin (Figure 5d, e). In diplotene, the bodies were still stained and disappeared in late diakinesis (Figure 5f). From metaphase I to telophase I, all chromosome bivalents were positively stained with silver nitrate (Figure 5g, h). With this staining procedure, all obtained bivalents seemed smaller than those obtained with other stain- ing procedures. Therefore, it was difficult to determine the exact locations of the nucleolar organizing regions on the chromosome. At the beginning of spermiogenesis, the round spermatids had a round silver nitrate stained mass at the periphery (Figure 5i). The nucleolar mass still presented at the middle of elongated spermatids (Figure 5j). When spermatids developed to be spermatozoas, the sperm heads were more strongly stained than other parts of their bodies (Figure 5k, l).

## Discussion

In this study, the meiotic chromosomes from testicular cells of *L. indicus* were analyzed. Like other insects of Heteroptera, *L. indicus* possess holocentric chromosomes (chromosome without localized centromeres). Therefore, it is impossible to describe the morphology of chromosomes (Papeschi and Bressa 2006). Two results of major interest were obtained in the present study. One was the presence of sex chromosomes and autosomal univalents in some testicular cells, and the other one was the clear presentation of mchromosome existence in the family Belostomatidae.

### Chromosome complement

The chromosome complement of *L. indicus* collected from Thailand, Loas, Myanmar, and Cambodia was 2n = 22A + neo-XY + 2m. In mitotic testicular cells, the two biggest chromosomes and the two smallest ones were obviously distinguished from the remaining autosomes. In mitotic cells stained with DAPI, the biggest chromosomes contained three DAPI-bright bodies, but their patterns were different. Prominently, one of the two biggest chromosomes had a DAPI-bright body at its end, while the other one had big DAPI-bright bodies in the middle region. The two biggest chromosomes were sex chromosomes, neo-X and neo-Y. It was difficult to point out which one was neo-X or neo-Y because their sizes were almost the same. However, in meiotic cells at diplotene stage, the neo-XY bivalent was clearly seen to be composed of two chromosomes joined together, with one side bigger than the other. It is likely that the bigger one may have been the neo-X and the smaller the neo-Y. On the smaller side of the neo-XY bivalent, two DAPI-bright bodies were observed; one DAPI-bright body was located at the end. On the bigger side, two DAPI-bright bodies were located down the end. Which chromosome is neo-X and which is neo-Y can be determined by comparing the DAPI-bright body pattern on the sex chromosomes in mitotic cells and in meiotic cells. Hence, in Figure 1a, the chromosome with a DAPI-bright body located at its one end might be neo-Y and the other might be neo-X.

To determine the location of nucleolar organizing regions in the chromosome complement of *L. indicus*, silver nitrate staining was carried out. In metaphase I through telophase I, silver nitrate positive signal was found in all chromosome bivalents except the mchromosome bivalent. The result was totally different from the previous reports of heteropteran insects. The location of nucleolar organizing regions in autosomal pairs was reported in some heteropteran insects such as *Nezara viridula* (Camacho et al. 1985), *Pachylis argentines* (Papeschi et al. 2003), *Spartocera fusca* (Cattani and Papeschi 2004), and *Limnogonus aduncus*, (Castanhole et al. 2008). Some species had nucleolar organizing regions located on sex chromosomes (Papeschi 1995). Therefore, the location of nucleolar organizing regions in *L. indicus* should be investigated further using advent techniques.

### Chromosome number change

The meiotic chromosome complement in testicular cells of *L. indicus* collected from Thailand, Loas, Myanmar, and Cambodia was 2n = 22A + neo-XY + 2m. In previous reports, the chromosome complement of *L. indicus* was 2n = 24A + neo-XY (Banerjee 1958; Bagga 1959; Jande 1959). However, it is unknown whether the m-chromosome existed in the *L. indicus* chromosome complement reported previously, because the original papers could not be obtained and only the chromosome formula was presented (Papeschi and Bidau 1985; Papeschi and Bressa 2006). If the m-chromosomes existed and were defined as two small autosomes, the chromosome number of *L. indicus* obtained in this study and that of the previous study were the same. However, according to Papeschi and Bressa (2006), the m-chromosomes were absent in *L. indicus*. Therefore, there are two likely possibilities to explain the difference in chromosome number obtained from this study with the previous reports. First, the giant water bugs used in this study and the one studied previously might not be the same species. It is impossible to prove this possibility, because the previous one was studied long time ago. However, it has been reported that the giant water bugs distributed in Southeast Asia belong to the same species, *L. indicus* (Perez-Goodwyn 2006). Genetic diversity studies of their mitochondrial genomes also support the report (unpublished data). Until now, the chromosome numbers of *Lethocerus* spp. have been studied in seven species (Papeschi and Bressa 2006). Three species, *L. annulipes, L. griseus, L. melloleitaoi* De Carlo, have the same chromosome number, 2n = 28 = 26A + XY. In the previous report, the chromosome number of *L. indicus* was 2n = 26 = 24A + neoX-neoY. Together with the analysis of chromosome numbers, it was also suggested that the ancestral chromosome number of insects belonging to the family Belostomatidae was 2n = 26 + XY (Papeschi and Bressa 2006). Therefore, *L. indicus* should have had the same chromosome number as the above three *Lethocerus* species. The reduction of its chromosome number from 2n = 26 + XY to 2n = 24 + neo-XY occurred by the translocation of the X and Y chromosomes to one pair of autosomes, resulting in the neo-X and neoY chromosomes (Nokkala and Nokkala 1999). The remaining three *Lethocerus* species, *L. americanus* Leidy (2n = 6+XY), *Lethocerus* sp. (2n = 2+ neoX-neoY) and *L. uhleri* (Montandon) (2n = ca. 30), had different chromosome numbers.

Second, the chromosome number difference might be due to the occurrence of chromosome polymorphism. The increase or decrease of chromosome numbers in insect species with holocentric chromosomes generally occurs by chromosome fragmentations and fusions (Ueshima 1979; Nokkala et al. 2006). The results of the chromosome arrangements are presented as chromosome polymorphism in populations prior to the chromosome change being fixed in a population. In Heteroptera, chromosome polymorphism has been reported in some species. For example, in a species of *Belostoma*, two karyotypic types were found, as the sex chromosomes showed to be polymorphic. In most individual males, the chromosome complement was 2n = 16 = 14 + XY, while some individuals had 2n = 17 = 14 +X_1_X_2_Y. It was proved that such chromosome polymorphism resulted from the breaking of the original X chromosome from the XY system into two unequal fragments, X_1_ and X_2_ (Papeschi 1996). In *Belostoma plebejum*, the chromosome polymorphism was the result of autosomal fusion, so the decrease of the chromosome number from 2n = 14 + XY to 2n = 13 +XY was reported (Papeschi 1994). In Homoptera, sex chromosome polymorphism was reported in *Cacapsylla* (Grozeva and Maryanska-Nadachowska 1995). The karyotype of *Cacapsylla mali* male samples collected from populations in Finland and Russia was 2n = 22 + neo-XY, while the karyotype of samples collected from a population in Poland was 2n = 20 + neo-X1X_2_Y. It is likely that chromosome polymorphism might occur in *L. indicus* populations from different areas because *L. indicus* is distributed from India to Southeastern Asia (Perez-Goodwyn 2006). The *L. indicus* samples used in this study and the samples investigated in the previous report might have been collected from populations in different locations.

### Origin of autosomal univalent chromosomes

In this study, the univalent chromosomes were found during the meiotic division in each testis, resulting in four cell types, A, B, C and D, with different univalent patterns. Type A cells with no univalent is the typical chromosome pattern of *L. indicus* used in this study. Type B and D cells contained two univalent autosomes since one autosomal pair did not form a bivalent. The presence of univalent autosomes was reported in some Heteroptera, such as *Myrmus miriformis* Fn. (Nokkala 1985), *Calocoris quadripunctatus* (Nokkala 1986), *Acanonicus hahni* (Papeshchi and Mola 1990), and *Largus rufipennis* (Mola and Papeschi 1993). The origin of univalent chromosomes is the result of homologous chromosomes asynapsis or desynapsis (White 1973). The distinction between asynapsis and desynapsis is quite difficult to observe because both processes occur at zygotene-pachytene, in which individual chromosomes are difficult to observe. In the case of asynapsis, pairing of homologous chromosomes is defective, so the chromosomes fail to do synapsis at zygotene. In the case of desynapsis, homologous chromosomes pair normally during zygotene, but chiasmata formation is defective, so the homologous chromosome undergoes desynaptic at late pachytene. John and Naylor (1961) suggested the occurrence of univalent chromosome formation might be caused by both genetics and environmental factors, but did not determine which one was the precise cause. For *A. hahni*, Papeshchi and Mola (1990) suggested univalent chromosomes did occur by desynapsi, because the univalent chromosomes were present in proximity and sometimes they were connected by a fine thread, which was the characteristic of desynapsis. In *L. rufipennis*, using the same characters as that of *A. hahni*, Mola and Papeschi (1993) suggested desynapsis to be the origin of autosomal univalents. With the above criteria, the origin of autosomal univalents found in type B and D cells may be desynapsis, because in some cells they were present close to each other. However, it was difficult to observe if autosomal univalents found in type B and D cells belonged to the same homologous couple, because most autosomes were similar in size.

### Origin of neo-XY chromosome univalents

Four sex chromosome systems have been found in Heteroptera. There are XY/XX, XO/XX, different multiple, and neo-sex chromosome systems (Nokkala and Nokkala 1999; Rebagliati et al. 2005; Papeschi and Bressa 2006; Maryanska-Nadachowska et al. 2008; Bressa et al. 2009; Grozeva et al. 2010). The first is the most commonly found system (71%), and the last is the most rare system, present in only seven species and subspecies of 1,600 species studied so far (0.4%), including *L. indicus* (Jande 1959). The neo-XY system of *L. indicus* was the result of the translocation between the X-Y chromosomes and a pair of autosomes. The behavior of neoXY chromosomes differed from that of the sex chromosome of other systems because the sex chromosomes, neo-X and neo-Y, formed a bivalent at prophase I, while the sex chromosomes of other systems formed univalents. In this study, the neo-X and neo-Y chromosomes forming a bivalent were found in type A and B cells, whereas in type C and D cells, the neo-X and neo-Y chromosomes were present as univalents. The origin of the neo-X and neo-Y univalents may be inferred to be a result of desynapsis because of three reasons: (1) they were present in some type C cells at diplotene and diakinesis stages, (2) univalent neo-X and neo-Y chromosomes were always close to each other, and (3) the fine thread linking the neo-X and neo-Y could be observed. The neo-X and neo-Y chromosomes have not been previously reported to form univalents.

### Behavior of chromosomes

In this study, in type A cells with no univalents, all chromosomes, including autosomes, sex chromosomes, and m-chromosomes, formed bivalents and divided prereductionally at anaphase I and segregated equaltionally at anaphase II. The meiotic behavior of autosomal bivalents did follow the rule of the order Heteroptera, in which the autosomal bivalents divide reductionally at anaphase I and segregate equaltionally at anaphase II (Ueshima 1979; White 1979). The general behavior of sex chromosomes during meiotic division of Heteropteran insects with XY/XX system instead follows the rule that the sex chromosomes are achiasmatic and form univalents. At anaphase I, the sex chromosomes segregate equationnally, in metaphase II they form a pseudobivalent, and at anaphase II they divide reductionally (Ueshima 1979; Suja et al. 2000). Since the sex chromosome system of *L. indicus* was the neo-XY system, the neo-X and neo-Y chromosomes did not follow this rule. On the contrary, in the neo-XY system, the sex chro- mosomes form a bivalent and divide exactly as autosomal bivalents, which divide prereductionally at meiosis I (White 1973). In the case of autosomal univalents, they behave in the same way as sex chromosome univalents do. Therefore, the behavior of univalent chromosomes in type B, C, and D cells were all the same, as they all divided equationally at anaphase I and segregated reductionally at anaphase II.

### The existence of m-chromosome

The presence of an m-chromosome pair was claimed to be a characteristic of the family Belostomatidae (Ueshima 1979). However, cytogenetics studies in 27 species of this family revealed the absence of m-chromosomes in their chromosome complement (Papeschi and Bidau 1985; Papeschi 1996; Papeschi and Bressa 2006). In this study, mitotic chromosomes of *L. indicus* showed two mchromosomes, so the existence of an mchromosome pair in this family is clear. The m-chromosomes were defined by their small size. The rule of m-chromosome behavior during meiosis I had been described as being usually achiasmatic chromosomes, not forming a bivalent, but in diakinesis they move closely to associate as ‘touch-and-go pairing’ in a form of pseudobivalent. In anaphase I, the m-chromosome pseudobivalent segregated reductionally, and then divided equationally in anaphase II (Ueshima 1979; Gonzalez-Garcia et al 1996; Suja et al. 2000; Papeschi and Bressa 2006). However, the behavior of mchromosomes in *L. indicus* represented another exception, because the m-chromosomes always appeared as a bivalent by associating distally during meiosis I in all cells. No univalent m-chromosome was observed. The presence of an m-chromosome bivalent behaving differently from the rule was reported in some species (Nokkala and Nokkala 1983; Nokkala 1986; Suja 2000). In *Saldula orthochila* and *S. saltatoria*, their m-chromosomes were always present as bivalents, exactly like *L. indicus*. These m-chromosomes were not considered to be true m-chromsomes. The appearance of an m-chromosome bivalent indicated that m-chromosomes behaved like other autosomes undergoing synapsis in prophase I to form a bivalent. Instead, the occurrence of m-chromosome univalents was the result of desynapsis, not of asynapsis (Nokkala and Nokkala 1983).

**Table 1.**
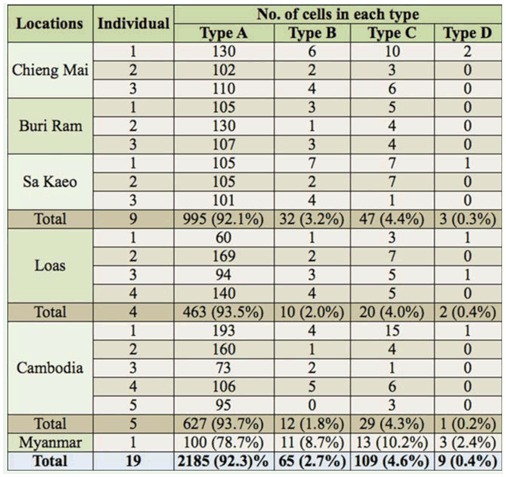
Frequence of each cell types found in testes of Lethocerus *indicus* males collected from Thailand (Chieng Mai, Buri Ram, and Sa Kaeo), Loas, Combodia, and Myanmar. Type A with no univalent, type B with autosomal univalents, type C with sex chromosome univalents, and type D with both sex chromosome and autosome univalents.
